# Controlled low central venous pressure after cardiopulmonary bypass reduces acute kidney injury following cardiac surgery: a randomized controlled trial

**DOI:** 10.3389/fmed.2026.1846773

**Published:** 2026-09-14

**Authors:** Lihai Chen, Lanxin Hu, Yi He, Siyu Kong, Jiacong Liu, Ke Ding, Chen Zhang, Duanqi Zhu, Yi Cheng, Hongwei Shi, Jifang Zhou, Yali Ge

**Affiliations:** 1 Department of Anesthesiology, Perioperative and Pain Medicine, Nanjing First Hospital, Nanjing Medical University, Nanjing, Jiangsu, China; 2 School of International Business, China Pharmaceutical University, Nanjing, Jiangsu, China

**Keywords:** acute kidney injury, cardiac surgery, cardiopulmonary bypass, central venous pressure, controlled low central venous pressure, venous congestion

## Abstract

**Background:**

Postoperative acute kidney injury (AKI) is a serious complication of cardiac surgery. This study aimed to determine whether targeting central venous pressure (CVP) readings of ≤10 mmHg using vasoactive drugs and reversing the Trendelenburg position was associated with a lower incidence of AKI than usual care in patients undergoing cardiac surgery requiring cardiopulmonary bypass (CPB).

**Methods:**

We conducted a randomized controlled trial. Patients were randomized in a 1:1 ratio to receive controlled low central venous pressure (CLCVP) or usual care. Patients in the CLCVP group received nitroglycerin and were placed in the reverse Trendelenburg position from 20 min after CPB until the end of surgery. The primary outcome was incidence of 7-day postoperative AKI defined by KDIGO criteria.

**Results:**

The trial included 505 subjects in the final analysis. There were fewer patients with postoperative AKI in the CLCVP group [114 (44.9%)] compared with the control group [150 (59. 8%)] [Absolute risk reduction (ARR): −14.9%; 95% confidence interval [CI]: (−23. 5%, −6. 3%); *P* = 0. 001]. Eight (3.1%) participants in the CLCVP group and 14 (5.6%) participants in the control group developed AKI of Stage 2 and above [ARR: −2. 4%; 95% CI: (−6. 0, 1. 1%); *P* = 0. 263]. This reduction was primarily driven by a lower incidence of Stage 1 AKI, whereas the incidence of Stage 2 or higher AKI did not differ significantly between groups. Median time-weighted CVP was lower in the CLCVP group (6.7 mm Hg vs. 8.1 mm Hg, *P* < 0. 001).

**Conclusion:**

This randomized clinical trial demonstrated that cardiac surgery patients with CLCVP exhibited lower CVP and fewer postoperative kidney injuries compared to those receiving usual care.

**Clinical Trial Registration:**

https://clinicaltrials.gov/study/NCT05855954, identifier NCT05855954.

## Introduction

Acute kidney injury (AKI) is a common and serious complication after cardiac surgery, which is associated with a worse prognosis, including longer hospitalization, elevated mortality and morbidity, and greater healthcare costs (1–3). The pathophysiological mechanisms underlying the development of AKI after cardiac surgery are complex. Venous congestion during cardiac surgery rather than hypotension is independently associated with postoperative adverse kidney events, for both AKI within 7 days postoperative and longer-term acute kidney disease (AKD) that develops between 8 and 90 days postoperative (4–6). Central venous pressure (CVP) is used as an indicator to quantify the degree of venous congestion, and high CVP may reflect severe venous congestion, contributing to decreased organ perfusion pressure and negatively affecting renal microcirculation (7–9).

Several preventive strategies have been investigated to reduce the incidence of cardiac surgery-associated AKI, including optimization of perioperative hemodynamic management, avoidance of nephrotoxic exposure, and early identification of high-risk patients. The PROTECTION trial and KDIGO-based care bundles have suggested potential benefits of structured preventive approaches, particularly among patients at increased risk of AKI. However, these strategies mainly focus on risk stratification and supportive management, and effective interventions directly targeting the underlying hemodynamic mechanisms of renal injury remain limited.

Venous congestion has recently been recognized as an important contributor to postoperative kidney dysfunction. Since elevated central venous pressure (CVP) may impair renal microcirculation and decrease renal perfusion pressure, interventions aimed at reducing venous congestion may represent a potential approach for renal protection. The controlled low central venous pressure (CLCVP) technique has been widely adopted in hepatectomy as a safe and effective strategy to reduce intraoperative blood loss (10, 11). CLCVP technology has been widely used in hepatectomy, but there are few studies on its application in cardiac surgery settings. Most scholars believe that it has no adverse effects on the kidneys, while some studies suggest that it has a certain protective effect on the kidneys, but the mechanism is unclear (12, 13). Therefore, whether CLCVP can reduce postoperative kidney injury after cardiac surgery requires further investigation. Actively targeting lower CVP levels using fluid restriction, the reverse Trendelenburg position, vasodilators, and other methods by anesthetics can significantly reduce major intraoperative bleeding events (13, 14). The CLCVP technique could potentially play a role in renal protection during cardiac surgery by easing venous congestion through reduced CVP levels.

We therefore carried out a controlled, randomized clinical trial to study the effect of the CLCVP technique on postoperative acute kidney complications after on-pump cardiac surgery. Specifically, we tested the hypothesis that CLCVP management reduces the incidence of postoperative AKI through lower intraoperative CVP levels.

## Methods

### Study design

This study was approved by the Institutional Review Board (IRB) of Nanjing First Hospital (Ethics No: KY20220805-04, August 5, 2022), and written informed consent was obtained from all subjects participating in the trial. This hospital is representative of its respective urban population and conducted >2,000 cardiac surgeries annually. The trial was registered prior to patient enrollment at clinicaltrials.gov (NCT05855954; Principal investigator: Yali Ge; Date of registration: May 10, 2023). The trial was conducted according to the Declaration of Helsinki (15). We followed the Consolidated Standards of Reporting Trials (CONSORT) reporting guideline (16).

### Selection criteria

(i) age ≥18 years; (ii) cardiac surgery requiring CPB; and (iii) elective procedures only.

Exclusion criteria included pre-existing renal insufficiency defined by the presence of abnormal preoperative serum creatinine ≥133 μmol/L and/or a preoperative diagnosis of renal insufficiency within 6 months of the preoperative period. Besides, patients with preoperative dialysis dependence within 60 days before surgery, off-pump heart surgery, prior kidney transplantation, and pregnancy were excluded.

### Sample size calculation

A retrospective study of 46,520 cases in critical condition from the United States found that the prevalence of any AKI was 60% when the urine output (UO) criteria were combined with SCR criteria (17). Referring to the study of CLCVP in liver surgery, we expect that CLCVP can reduce the incidence of AKI after cardiac surgery by at least 15%. A total of 462 patients (231 patients per group) were required to achieve 90% power at a two-sided α level of 0.05. Considering a potential 10% rate of all-cause loss to follow-up or invalid data, we planned to recruit at least 254 patients in each group.

### Randomization and masking

After providing written informed consent, eligible patients were randomized using a secure web-based electronic central randomization system. An independent statistician performed block randomization via computer-generated random numbers with block sizes of 3, 6, or 9 to ensure balanced allocation concealment. Eligible patients were randomly assigned in a 1:1 ratio to either the CLCVP group (intervention group) or the usual care group (control group)**.** Due to the nature of the intervention, treating anesthesiologists could not be blinded to group allocation. However, participants, outcome assessors, and statistical analysts remained blinded throughout the study. The investigators remained blinded to the treatment allocation and did not access the study data until the database was locked and treatment allocation was revealed.

### Procedures

During the pre-anesthesia consultation, patients were provided with a comprehensive explanation of the research protocol. Before entering the operating room, patients underwent monitoring procedures, including invasive blood pressure (BP) measurement, electrocardiogram (ECG), pulse oxygen saturation assessment, bispectral index (BIS) monitoring, and central venous pressure (CVP) monitoring. The CVP transducer was positioned at the midpoint of the right atrium, specifically at the fourth intercostal level of the axillary midline in the supine position. Anesthesia induction involved the administration of propofol, sufentanil, and cisatracurium, followed by maintenance with propofol, remifentanil, and cisatracurium. Conforming to recent literature (2, 18), the management criteria for cardiopulmonary bypass (CPB) were as follows: maintaining a mean arterial blood pressure (MAP) of 60–70 mm Hg, utilizing non-pulsatile CPB, α-stat acid-base management for carbon dioxide tension regulation, maintaining hematocrit levels between 25% to 30%, keeping blood glucose levels below 200 mg/dL, and incorporating arterial line filters. Systemic anticoagulation was achieved using unfractionated heparin administered at a dose of 300 IU/kg before initiation of cardiopulmonary bypass (CPB). Activated clotting time (ACT) was monitored throughout the CPB period and maintained above 480 s to ensure adequate anticoagulation. After completion of CPB and separation from bypass, anticoagulation was reversed with protamine sulfate at a protamine-to-heparin ratio of 1:1.

Approximately 20 min after separation from CPB, the CLCVP technique was initiated, involving the administration of nitroglycerin and the adjustment of body position until the conclusion of the surgery. Patients were placed in a reverse Trendelenburg position with the head elevated by 10°. Nitroglycerin was administered incrementally, starting at a dose of 0.25–1 μg/kg/min, with the option to increase by 0.25 μg/kg/min every 3 min based on maintaining an acceptable MAP. The target CVP value for the study was set at below 10 mmHg based on prior research (4). If a patient's initial CVP was below 10 mmHg, intravenous nitroglycerin was maintained at 0. 25 μg/kg/min until the end of surgery, while other aspects of the CLCVP technique were withheld. Standard vasoactive medications such as norepinephrine were administered as needed to ensure a MAP ≥60 mmHg. The control group received standard care without intervention.

### Intention-to-treat population

All randomized patients with valid hemodynamic data and follow-up information were included in the modified intention-to-treat (mITT) population. The modified intention-to-treat (mITT) population was prespecified as all randomized patients who met the eligibility criteria, underwent cardiac surgery with CPB, and had complete valid hemodynamic data and follow-up information available.

Compared with the strict ITT principle, the only modification in our mITT approach was the exclusion of patients with missing critical primary endpoint data (hemodynamic parameters or follow-up), which was prespecified in our sample size calculation (anticipating a 10% rate of invalid or missing data, we deliberately inflated the enrollment to maintain adequate power).

Importantly, patients with pre-existing renal dysfunction were excluded at the screening stage per the selection criteria rather than being *post-hoc* removed from the ITT set. Both primary and secondary endpoints were analyzed using this full mITT population, which aligns with the prospective analytic plan.

### Primary outcomes

The primary endpoint of the study was the occurrence of acute kidney injury (AKI) within 7 days post-surgery. We operationalized postoperative AKI in accordance with the Kidney Disease: Improving Global Outcomes (KDIGO) Clinical Practice Guideline for Acute Kidney Injury, defined as an absolute increase in serum creatinine of ≥0. 3 mg/dL (≥26.5 mmol/L) within 2 days or a 1.5-fold increase in serum creatinine from baseline within 7 days, or UO <0.5 mL/kg/h for more than 6 h in the ICU after surgery (19). UO was measured using consecutive hourly readings. Additionally, sensitivity analyses were conducted using various AKI definitions, including mean hourly UO measured over 6, 12, and 24-hour periods, as well as analyses excluding UO measurements to simulate observational trials where quantifying UO may be impractical.

### Secondary outcomes

Secondary outcomes included the incidence of AKI of stage 2 and above within 7 days; *de novo* renal replacement therapy (RRT) initiation; changes in venous pressure (CVP) and mean arterial pressure (MAP); in-hospital death; intensive care unit (ICU) admission; and hospital length of stay (LOS).

### Relevant clinical variables

Patient demographic and clinical characteristics, including age, sex, the presence of comorbidities, and the types of surgery, were directly abstracted from the medical record. Laboratory results such as creatinine and hemoglobin were captured using the inpatient laboratory information system (LIS). The perioperative use of antihypertensive agents, inotropic drugs, vasopressors, diuretics, anesthesia medication, and antiarrhythmic drugs was extracted from electronic health records (EHRs).

### Statistical methods

Patient-level demographic and clinical characteristics were compared between the CLCVP and control groups. Continuous variables were presented as mean ± SD or median (interquartile range, IQR), and categorical variables as *n* (%). The standardized effect size between two arms was estimated using standardized mean difference (student's *t*-test statistic), standardized median difference (Wilcoxon Z statistic), and square root of *χ*2 test (or Fisher exact test if the produced matrices contained cells with expected counts <5) for comparisons between continuous and categorical variables as appropriate.

The primary efficacy analysis included all randomized patients and was performed according to the intent-to-treat principle, i.e., all patients were analyzed according to their randomization status. For the logistic regression analyses, complete-case analysis regarding the included covariates was included. No imputation of the data was performed.

We estimated the relative risk (RR) reduction and the absolute risk reduction, including 95% confidence intervals (CIs), for the occurrence of AKI between the two study groups. The effect of CLCVP on the incidence of post-operative AKI was summarized using odds ratios (OR) in logistic regression models. A *P*-value <0.05 was considered statistically significant. All analyses were performed in R version 4. 0. 3 (R Foundation for Statistical Computing, Vienna, Austria).

## Results

### Study cohort and patient characteristics

A total of 976 patients were assessed for eligibility from 15 May 2023 to 29 November 2023, and 531 underwent randomization, with 266 assigned to the CLCVP group and 265 to usual care. 11 patients did not receive surgery, and 15 subjects received off-pump CABG; they were not included in the analysis. There were no patients lost to follow-up. Data from 505 eligible subjects were included in the final analysis (Figure 1).

**Figure 1:**
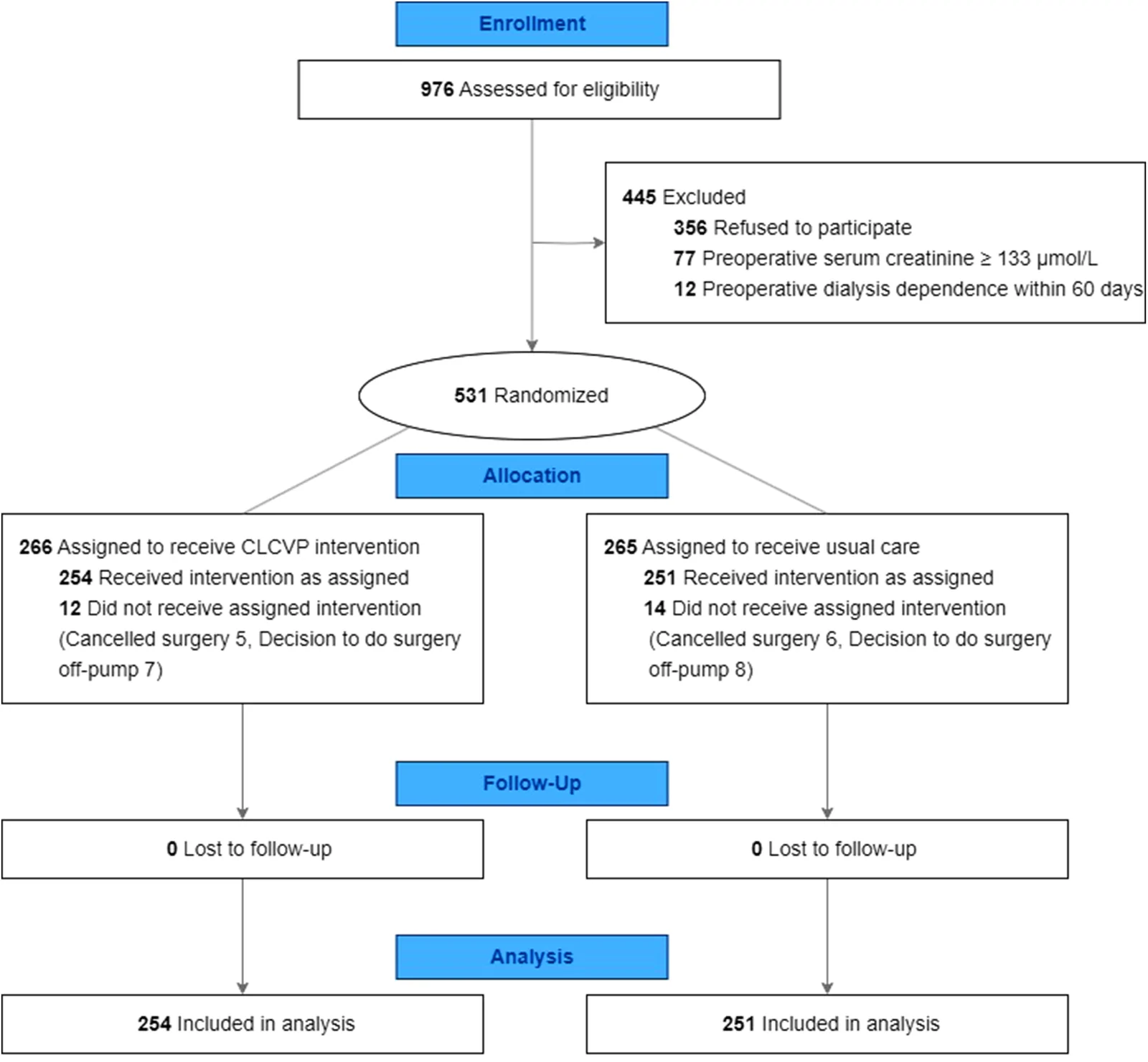
Trial flowchart. CLCVP, controlled low central venous pressure.

Patient baseline demographic and clinical characteristics, including operation types and surgery-related characteristics, are displayed in Table 1. Baseline subject characteristics were similar between groups. Most subjects underwent valve replacement surgery-only, followed by CABG-only and combined CABG-valve procedures. There was no difference in preoperative hemoglobin level and estimated glomerular filtration rates (eGFR) between groups.

**Table 1:** Demographics, surgery-related, and intraoperative characteristics of study subjects.

Characteristics	Control	CLCVP
	*N* = 251	*N* = 254
Baseline demographics and comorbidities		
Age at surgery, years [median (IQR)]	65 (57, 71)	63 (56, 71)
Female, *n* (%)	91 (36.3)	114 (44.9)
Body weight, kg [median (IQR)]	64.0 (55.0, 72.5)	65.0 (58.0, 73.0)
BMI (median (IQR)	23.8 (21.5, 25.7)	24.3 (22.1, 26.3)
ASA Grade		
Grade 3, *n* (%)	246 (98.0)	254 (100.0)
Grade 4, *n* (%)	5 (2.0)	0 (0.0)
Preoperative comorbidities		
Anaemia^a^, *n* (%)	54 (21.5)	69 (27.2)
Hypertension, *n* (%)	124 (49.4)	129 (50.8)
Diabetes, *n* (%)	32 (12.7)	35 (13.8)
Atrial fibrillation, *n* (%)	41 (16.3)	31 (12.2)
Chronic obstructive pulmonary disease, *n* (%)	1 (0.4)	0 (0.0)
Baseline laboratory results		
Hb level, g L^−1^ [median, (IQR)]	132.0 (121.0, 142.0)	130.0 (118.3, 141.0)
Glucose level, mmol L^−1^ [median (IQR)]	5.7 (5.0, 7.3)	5.7 (5.0, 7.2)
Estimated glomerular filtration rate^b^, mL min^−1^ 1.73m^−2^ [median (IQR)]	89.5 (77.8, 97.7)	93.1 (83.7, 100.9)
Surgery types		
Valve replacement surgery-only, *n* (%)	120 (47.8)	118 (46.5)
CABG-only, *n* (%)	81 (32.3)	100 (39.4)
Combined CABG-valve procedure, *n* (%)	46 (18.3)	33 (13.0)
Other, *n* (%)	4 (1.6)	3 (1.2)
Surgery-related characteristics		
Duration of surgery, min [median (IQR)]	260 (230, 320)	260 (225, 290)
Length of anaesthesia, min [median (IQR)]	295 (256, 350)	290 (255, 325)
CPB duration, min [median (IQR)]	113 (87, 144)	107 (86, 136)
Intraoperative pRBC transfusion, mL [median (IQR)]	1,250 (1,000, 1,590)	1,200 (1,000, 1,500)
Intraoperative colloid infusion, mL [median (IQR)]	1,000 (1,000, 1,500)	1,000 (1,000, 1,500)
Intraoperative crystalloid infusion, mL [median (IQR)]	1,500 (1,000, 1,500)	1,000 (1,000, 1,500)
Intraoperative urine output, mL (median (IQR)	1,100 (725, 1,500)	1,050 (735, 1,477)
Intraoperative blood losses, mL (median (IQR)	1,200 (1,000, 1,325)	1,150 (1,000, 1,300)
Intraoperative medication usage		
Intraoperative nitroglycerin usage, *n* (%)	11 (4.4)	254 (100.0)
Intraoperative nitroglycerin dosage, mg [median (IQR)]	0.0 (0.0, 0.0)	1.8 (1.1, 2.7)
Intraoperative noradrenaline usage, *n* (%)	70 (27.9)	68 (26.8)
Intraoperative phenylephrine usage, *n* (%)	247 (98.4)	251 (98.8)
Intraoperative epinephrine usage, *n* (%)	32 (12.7)	6 (2.4)
Intraoperative ephedrine usage, *n* (%)	136 (54.2)	124 (48.8)
Intraoperative dobutamine usage, *n* (%)	44 (17.5)	30 (11.8)
Intraoperative dopamine usage, *n* (%)	21 (8.4)	9 (3.5)
Intraoperative hypophysine usage, *n* (%)	15 (6.0)	7 (2.8)
Intraoperative metaraminol usage, *n* (%)	2 (0.8)	0 (0.0)
Haemodynamic measures^c^		
Time-weighted average CVP, mmHg [median (IQR)]	8.1 (6.0, 10.5)	6.7 (5.0, 9.1)
Cumulative duration for CVP ≥10 epochs, min [median (IQR)]	15.0 (1.0, 46.0)	11.0 (0.0, 30.0)
Time-weighted average MAP, mmHg [median (IQR)]	72.3 (67.7, 78.4)	73.2 (69.1, 77.8)

CLCVP, controlled low central venous pressure; IQR, interquartile range; BMI, body mass index; Hb, haemoglobin; CABG, coronary artery bypass graft; CPB, cardiopulmonary bypass; pRBC, packed red blood cell; SD, standard deviation; CVP, central venous pressure; MAP, mean arterial pressure; AUC, area under the curve.

a Anaemia is defined by WHO (preoperative haemoglobin value of <130 g/L for men and <120 g/L for women).

b Estimated glomerular filtration rate is calculated by the 2021 CKD-EPI equation.

c Post-CPB.

### Intraoperative management and hemodynamic changes

Intraoperative hemodynamic data and medication administration are shown in Table 1. Mean surgery (260 min vs. 260 min, *P* = 0.112) and CPB duration (107 min vs. 113 min, *P* = 0. 092) was similar between groups (260 min vs. 260 min, *P* = 0. 112). There was no significant difference in the amount of fluid infusion, packed red blood cell (pRBC) transfusion, or urine output. With the exception of intraoperative nitroglycerin (CLCVP 100% vs. control group 4. 4%, *P* < 0. 001, epinephrine (CLCVP 2.4% vs. control group 12. 7%, *P* < 0. 001) and dopamine (CLCVP 3.5% vs. control group 8. 4%, *P* = 0. 035) administration, other utilization patterns of intraoperative vasopressin and vasodilator agents were generally comparable. There were significant differences between the intervention arms with respect to the CVP-related measures while demonstrating comparable intraoperative MAP levels (Table 1). Detailed hemodynamic variations on CVP, MAP, and mean perfusion pressure (MPP) across two arms are shown in Figure 2, stratified by relative to CPB timing and divided into pre-CPB, during CPB, and post-CPB periods.

**Figure 2:**
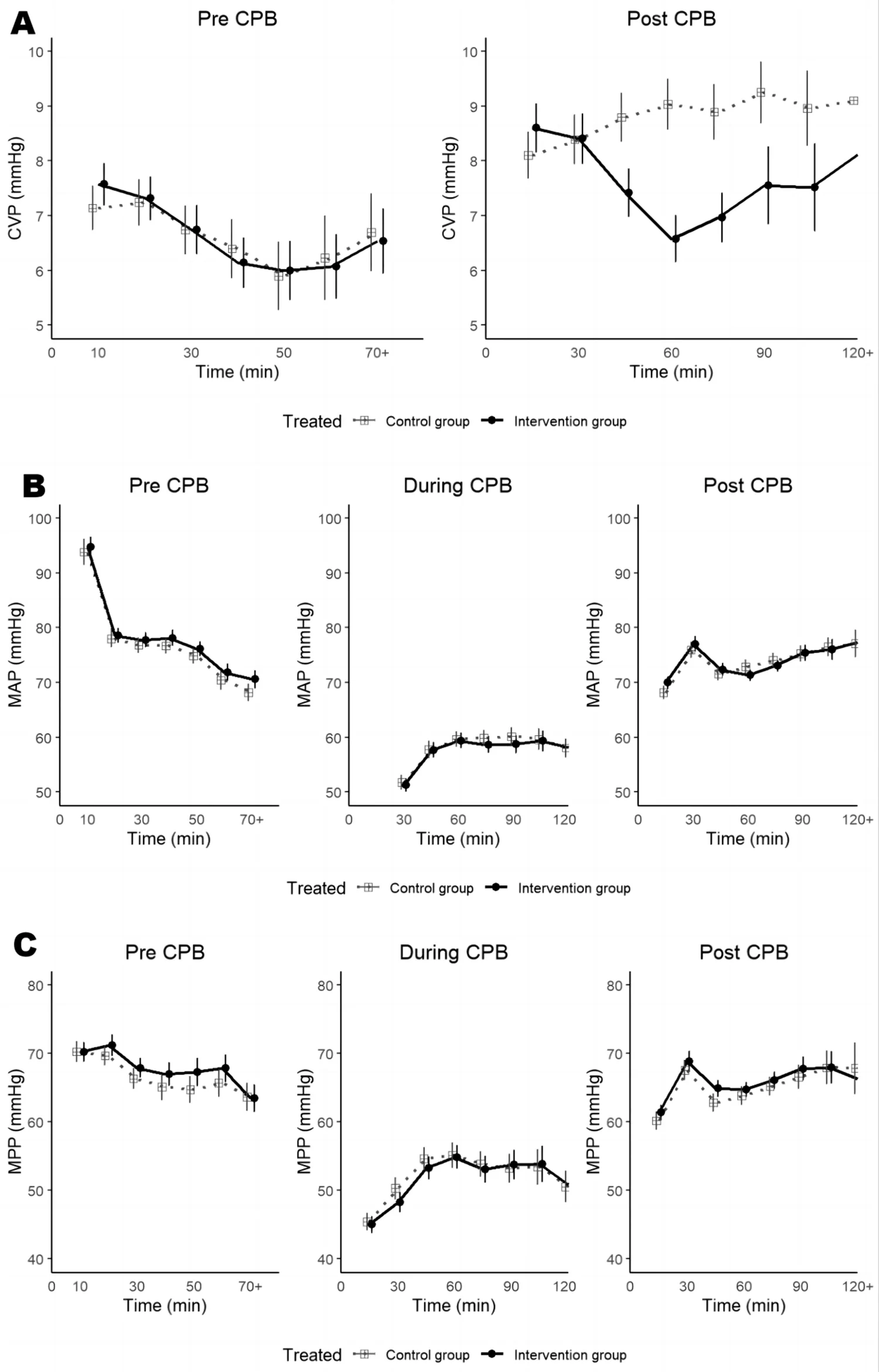
Perioperative hemodynamic changes in time-weighted mean central venous pressure (CVP) **(A)**, mean arterial pressure (MAP) **(B)**, and mean perfusion pressure (MPP) **(C)** among eligible study subjects. CPB, cardiopulmonary bypass; CVP, central venous pressure; MAP, mean arterial pressure; MPP, mean perfusion pressure.

### Postoperative clinical outcome

AKI occurred in 114/254 (44.9%) subjects randomized to CLCVP, compared with 150/251 (59.8%) randomized to usual care (ARR: −14). 9%; 95% confidence interval [CI]: (−23. 5%, −6. 3%); *P* = 0. 001), as defined by KDIGO serum creatinine criteria and UO_cont_ measurements. The reduction in postoperative AKI was primarily attributable to a lower incidence of Stage 1 AKI (51.2% vs. 41.7%), whereas the incidence of Stage 2 or higher AKI remained low and did not differ significantly between the two groups (5.6% vs. 3.1%, *P* = 0.263) (Table 2). The perioperative changes in serum creatinine level over time were similar between groups (eFigure 2). Cumulative incidence curves of AKI across treatment a5rms are shown in Figure 3.

**Table 2:** Postoperative outcomes.

Characteristics	Control	CLCVP	Absolute risk difference (95% CI)	*P*-value
	*N* = 251	*N* = 254		
**Presence of CSA-AKI** **(Accounting for SCr and UO_cont_)**	150 (59.8)	114 (44.9)	−14.9% (−23.5%, −6.3%)	0.001
CSA-AKI Stage 1, *n* (%)	136 (51.2)	106 (41.7)	−12.5% (−21.1%, −3.8%)	0.007
CSA-AKI Stage 2, *n* (%)	12 (4.8)	6 (2.4)	−2.4% (−5.6%, 0.8%)	
CSA-AKI Stage 3, *n* (%)	2 (0.8)	2 (0.8)	−0.01% (−1.5%, 1.6%)	
Secondary outcomes				
RRT initiation, *n* (%)	1 (0.4)	0 (0.0)	−0.4% (−1.2%, 0.4%)	0.995
CSA-AKI Stage 2+, *n* (%)	14 (5.6)	8 (3.1)	−2.4% (−6.0%, 1.1%)	0.263
Length of stay, days [median (IQR)]	17 (15, 21)	17 (14, 19)	0 (−2, 0)	0.030
ICU stay, hours [median (IQR)]	20 (17, 43)	19 (18, 36)	−1 (−2, 0)	0.084
Inpatient death, *n* (%)	4 (1.6%)	3 (1.2%)	−0.4% (−2.5%, 1.6%)	0.723
30-day all-cause mortality, *n* (%)	4 (1.6%)	2 (0.8%)	−0.7% (−2.6%, 1.1%)	0.448

CLCVP, controlled low central venous pressure; CSA-AKI, cardiac surgery-associated acute kidney injury; CI, confidence interval; SCr, serum creatinine; UO, urine output; IQR, interquartile range; RRT, renal replacement therapy; ICU, intensive care unit; ARR, absolute risk reduction. Length of anesthesia: the time from induction of general anesthesia to completion of anesthesia management before ICU transfer.

**Figure 3:**
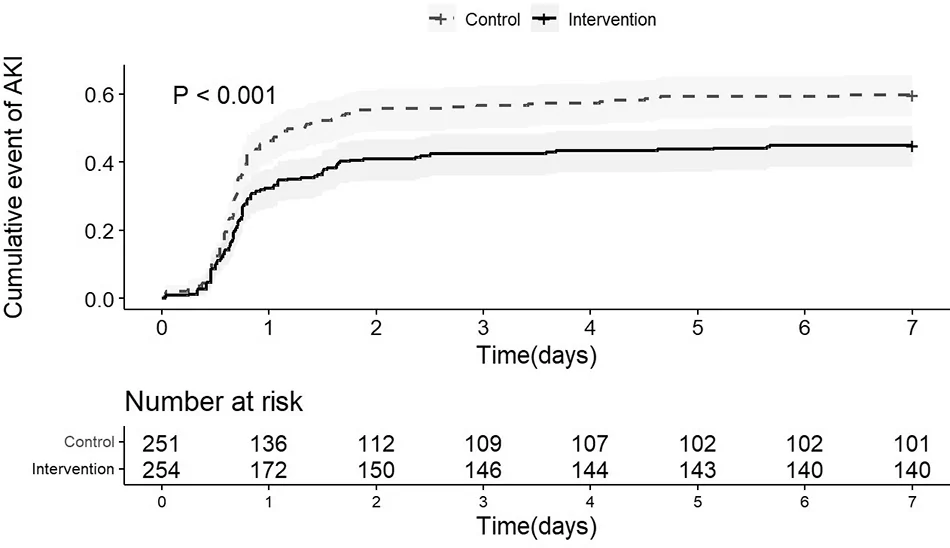
Cumulative incidence curve of acute kidney injury (AKI).

### Secondary outcomes

Renal replacement therapy was initiated in a patient from the control group, and none among patients undergoing CLCVP (0% *vs.* 0%). 4%, ARR: −0. 4%; 95% CI: (−1. 2%, 0. 4%); *P* = 0. 995). All-cause 30-day mortality was similar between subjects allocated to CLCVP [2/254 (0.8%)] or standard care [4/251 (1. 6%)], ARR: −0. 7%; 95% CI: (−1. 1%, 2. 6%); *P* = 0. 448. Besides, ICU and hospital lengths of stay were also evaluated in patients in the CLCVP group (Table 2).

### Subgroup analyses

Prespecified subgroup analyses demonstrated comparable rates of primary outcomes between subgroups, consistent with the findings from the full analytical set (Figure 4).

**Figure 4:**
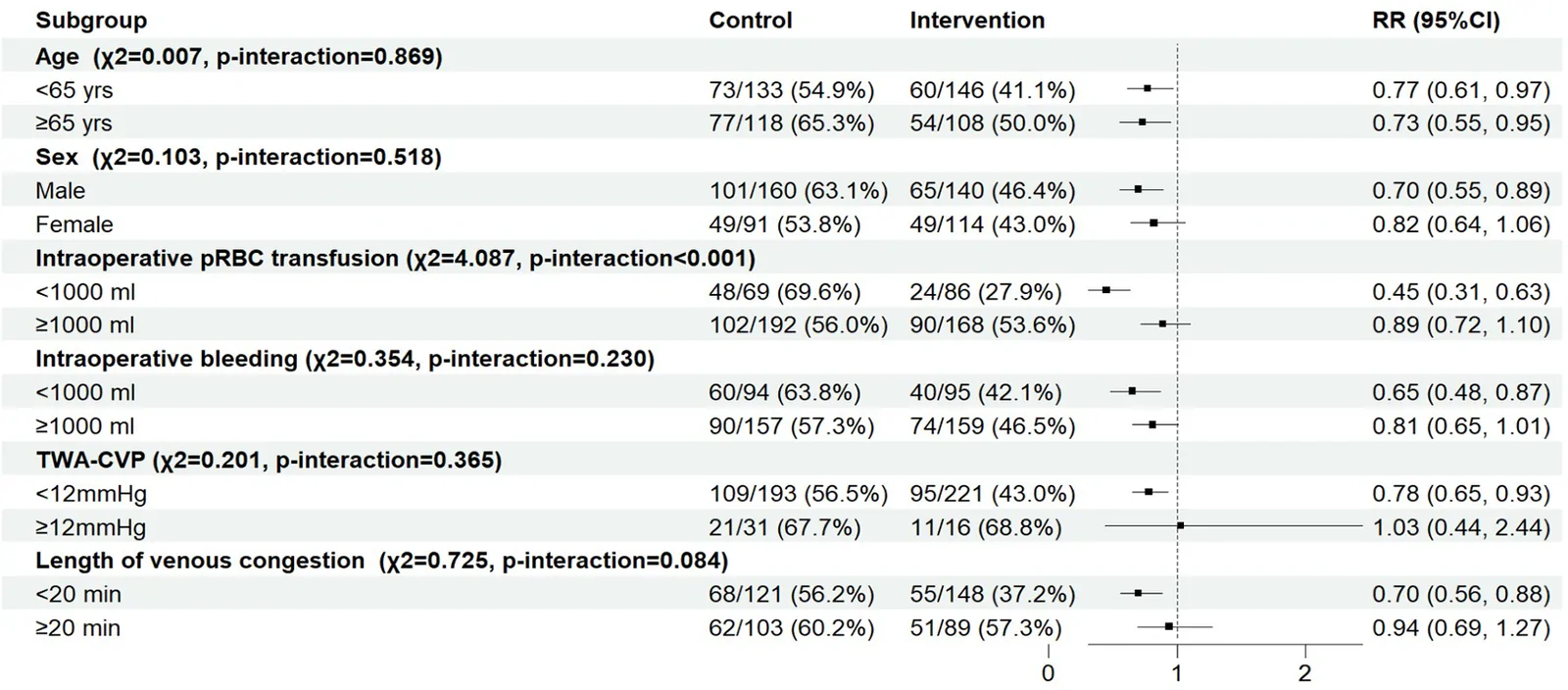
Forest plots on subgroup analysis. RR, risk ratio; pRBC, packed red blood cell; TWA, time-weighted average; CVP, central venous pressure.

### Sensitivity analyses

To compare the impact of using UO on the incidence of AKI, we further conducted sensitivity analysis. We found that when UO was included in the definition of AKI, the incidence of AKI was different between the control group and the intervention group, while no significant difference was found in the incidence of AKI between groups when UO was not included (eTable 2).

## Discussion

### Main findings

Our study revealed that the CLCVP technique significantly reduces the incidence of AKI at 7 days postoperatively compared to usual care (59.8% vs. 44. 9%), particularly evident on the first postoperative day. Importantly, this benefit was primarily driven by a reduction in Stage 1 AKI, whereas no statistically significant difference was observed for Stage 2 or higher AKI.The CLCVP group exhibited enhanced management of venous congestion and lower CVP levels compared to those receiving usual care. Furthermore, our findings indicate that the efficacy of the CLCVP technique is contingent upon the extent of CVP reduction, exhibiting a dose-response relationship. These results suggest that even transient regulation of CVP and amelioration of venous congestion following CPB can effectively safeguard renal function during the early postoperative phase.

### Strengths and mechanistic explanation of the renoprotective effects of CLCVP

The etiology of AKI post-cardiac surgery is multifaceted and intricate (20, 21). While hypotension and venous congestion are recognized as risk factors for AKI following cardiac procedures (22–24), the efficacy of vasopressor drugs in improving postoperative outcomes remains uncertain (25). This ambiguity arises from the potential of vasopressor drugs to elevate systemic vascular resistance, potentially compromising organ perfusion by diminishing microvascular blood flow. Thus far, no intervention has conclusively demonstrated protective effects against AKI by prospectively modulating hemodynamic changes during cardiac surgery. Improving venous congestion and consequently renal perfusion may offer a more promising therapeutic approach. However, distinguishing intraoperative hypotension from venous congestion in observational studies proves challenging due to their intrinsic interrelation and confounding by unmeasured covariates such as renal microperfusion, fluid volume status, and vascular resistance.

CVP serves as an indicator quantifying the extent of venous congestion, with elevated CVP potentially reflecting severe venous congestion leading to diminished organ perfusion pressure (5, 8, 26). Although the median time-weighted average CVP values in both groups remained within the clinically accepted range (8.1 vs. 6.7 mmHg), even a modest reduction in venous pressure may improve renal perfusion pressure and alleviate renal venous congestion. This finding suggests that renal function may be sensitive to relatively small changes in CVP, even within the conventionally normal range, particularly during the vulnerable post-CPB period. In contrast to previous studies, our trial uniquely investigated intraoperative hemodynamic variations with and without targeted CVP interventions. Our results affirm, in line with prior retrospective studies utilizing MAP and CVP measurements, that venous congestion, rather than hypotension, appears to be causative in renal function impairment postoperatively.

The randomized design of our trial, coupled with meticulous process documentation and lower-than-anticipated attrition rates, enhances the accuracy and reproducibility of our findings. By precisely documenting the dynamic variability of CVP, MAP, and MPP to elucidate their respective effects on renal outcomes, we assessed the renoprotective effects attributable to the CLCVP approach in cardiac surgery patients. The Numbers Needed to Treat (NNT) to prevent a case of AKI and Stage 2+ AKI were 7 and 2, respectively. Although post-CPB MAP was lower in the intervention group, this was counterbalanced by reduced CVP measurements and potentially improved renal perfusion secondary to nitroglycerin administration, as evidenced by enhanced MPP levels in the intervention group (27, 28).

Implementation of the CLCVP technique in cardiac surgery has demonstrated safety. Notably, in the context of liver surgery, the CLCVP approach has proven effective in reducing bleeding without increasing adverse surgical events (10, 14). However, it is imperative to underscore the substantial divergence between the CLCVP methodology in cardiac versus liver surgery. Unlike liver surgery, where the primary focus often centers on limiting intraoperative bleeding by maintaining CVP below 5 mmHg through volume restriction, our approach adopts a distinct strategy. We advocate gentle control of CVP below 10 mm Hg, as per our previous research (4), achieved through a combination of reverse Trendelenburg positioning (head elevated by 10°) and judicious nitroglycerin use to alleviate venous congestion. Our findings strongly suggest that this tailored approach not only safeguards renal function but also reduces the incidence of postoperative AKI in cardiac procedures. In addition, subgroup analyses should be interpreted cautiously. Although several interaction *p*-values appeared statistically significant, these subgroup analyses were exploratory and not specifically powered for interaction testing. Therefore, the possibility of a type I error cannot be excluded, and these findings should be considered hypothesis-generating rather than definitive evidence.

Venous congestion precipitates reduced renal blood perfusion and elevates tubular lumen pressure, diminishing trans-glomerular pressure gradients. This triggers alterations in glomerular feedback mechanisms, such as the renal angiotensin-aldosterone system, inducing systemic and renal inflammation, heightened endothelial permeability, and increased sympathetic outflow (5, 29). Underlying pathophysiological mechanisms encompass decreased renal blood flow and upregulation of NF-*κ*B signaling, which synergistically augment neutrophil adhesion to activated endothelial cells (30). Nitroglycerin's vasodilatory effects, primarily targeting veins, contribute to reduced venous return and subsequent preload reduction, aligning with the objectives of the CLCVP approach. Notably, in porcine models, nitroglycerin may exacerbate late nitrogen monoxide (NO)-mediated oxidative stress, while its effect could be partly attributed to NO-mediated microvascular activities (31, 32). Nonetheless, sildenafil-induced alterations in NO activity demonstrated limited therapeutic efficacy in adult cardiac surgery patients (33). Lastly, the time-varying effects of vasoactive agents and cumulative doses of both noradrenaline and nitroglycerin are contingent upon individual responsiveness to pharmacotherapy.

### Limitations

The primary limitation of the trial was the lack of blinding among treating anesthesiologists, potentially leading to bias toward superior clinical management in the CLCVP group. Although the CVP transducer was positioned according to a standardized protocol at the level of the right atrium, CVP measurement is inherently sensitive to transducer leveling, and small deviations in transducer position may have influenced the recorded CVP values. Therefore, measurement bias cannot be completely excluded. Secondly, accurate measurement of urine output was only available in ICU settings, potentially resulting in underdocumentation of AKI after transfer from the cardiac ICU to general surgical wards. In sensitivity analyses excluding the urine output criterion, the between-group difference in AKI incidence was attenuated and no longer statistically significant, suggesting that the observed benefit was partly driven by urine output-based AKI definitions. Thirdly, the wide variation in nitroglycerin doses administered in the CLCVP group was related to the goal-targeted CVP control strategy and individualized adjustment of hemodynamic management, which may limit standardization. In addition, because the intervention combined reverse Trendelenburg positioning with titrated nitroglycerin administration, the independent effects of each component could not be determined.

Fourthly, differences in intraoperative vasoactive medication use, such as epinephrine and dopamine, may have influenced outcomes, although achieving identical dosing between groups was impractical due to individualized hemodynamic requirements. Additionally, postoperative management variables, including fluid therapy, postoperative vasoactive support, potential nephrotoxic medication exposure, and time to extubation, were not systematically collected and could not be fully evaluated. Although all patients received standardized postoperative care according to institutional protocols, these unmeasured factors may have influenced postoperative outcomes. Finally, due to the single-center design and limited sample size, the generalizability of our findings may be limited. Future multicenter prospective randomized controlled trials with comprehensive perioperative data collection are warranted to further validate the renal effects of CLCVP.

### Clinical relevance

The trial yields three novel findings. Firstly, the CLCVP intervention aimed at achieving lower targeted post-CPB CVP-induced mild and transient intraoperative hypotension, corrected by vasopressors, with statistically nonsignificant rates of adverse acute renal complications, thereby supporting the causal role of venous congestion over intraoperative hypotension. Secondly, even short-term control of CVP and reduction of venous congestion after CPB demonstrate efficacy in protecting renal function during the early postoperative period. Thirdly, the predefined and registered primary endpoint of AKI, defined by the KIDGO guidelines using UO as a continuous measurement, underscores the importance of methodological rigor in data analysis.

## Conclusions

For adult patients undergoing cardiac surgery requiring CPB, CLCVP reduces the incidence of acute renal dysfunction in the 7-day postoperative period, warranting further investigation in a broader population. This justifies advancing to a large multicenter, open-label, prospective, randomized study to conclusively determine whether the renoprotective effect of CLCVP in cardiac surgery patients correlates with improved long-term renal outcomes compared to usual care.

## Data Availability

The data analyzed in this study is subject to the following licenses/restrictions: The data that support the findings of this study are not publicly available due to privacy or ethical restrictions but are available from the corresponding author upon reasonable request. Requests for access to the data should be directed to ge_yl@163.com.
