# Supplementary material for: Controlled low central venous pressure after cardiopulmonary bypass reduces acute kidney injury following cardiac surgery: a randomized controlled trial
Source: Front Med (Lausanne). 2026 Sep 14;13:1846773. doi: 10.3389/fmed.2026.1846773 (PMC13617087; doi:10.3389/fmed.2026.1846773)
Supplement: Supplementary file 1 [file Datasheet1.docx]

**TITLE**

Effect of Controlled Low Central Venous Pressure on Postoperative Acute Kidney Injury in Cardiac Surgery: A Randomized Clinical Trial

List of supplements.

**eTable 1.** KDIGO staging system for acute kidney injury.

**eTable 2.** Sensitivity analyses AKI case ascertainment criteria using mean urine output and without urine output values.

**eTable 3.** Discordance of CSA-AKI based on criteria with and without urine output.

**eFigure 1.** Incidence of acute kidney injury.

**eFigure 2.** Perioperative changes of serum creatinine relative to baseline values.

**eFigure 3.** Postoperative changes of urine output among study subjects.

**eTable 1.** KDIGO staging system for acute kidney injury.

| **Stage** | **Serum creatinine** | **Urine output** |
| --- | --- | --- |
| 1 | 1.5-1.9 times baseline, or  $\geq$0.3mg/dl increase | <0.5 ml/kg/h for 6-12h |
| 2 | 2.0-2.9 times baseline | <0.5 ml/kg/h for $\geq$12h |
| 3 | $\geq$3.0 times baseline, or  Increase in serum creatinine to $\geq$4.0 mg/dl, or  Initiation of renal replacement therapy | <0.3 ml/kg/h for $\geq$24h, or  Anuria for $\geq$12h |

Abbreviations: KDIGO, Kidney Disease: Improving Global Outcomes.

**eTable 2.** Sensitivity analyses AKI case ascertainment criteria using mean urine output and without urine output values.

| **Characteristics** | **Control** | **CLCVP** | ***P*-value** |
| --- | --- | --- | --- |
|  | **N=251** | **N=254** |  |
| **Presence of AKI (Accounting for both SCr and UO_mean_)** | 150 (59.8) | 114 (44.9) | 0.001 |
| No CSA-AKI | 101 (40.2) | 140 (55.1) | 0.007 |
| CSA-AKI Stage 1 | 136 (51.2) | 106 (41.7) |  |
| CSA-AKI Stage 2 | 12 (4.8) | 6 (2.4) |  |
| CSA-AKI Stage 3 | 2 (0.8) | 2 (0.8) |  |
| **Presence of AKI (Based on Scr without accounting for UO)** | 60 (23.9) | 47 (18.5) | 0.169 |
| No CSA-AKI | 191 (76.1) | 207 (81.5) | 0.512 |
| CSA-AKI Stage 1 | 51 (20.3) | 39 (15.4) |  |
| CSA-AKI Stage 2 | 7 (2.8) | 6 (2.4) |  |
| CSA-AKI Stage 3 | 2 (0.8) | 2 (0.8) |  |

Abbreviations: CLCVP, controlled low central venous pressure; AKI, acute kidney injury; SCr, serum creatinine; UO, urine output; CSA-AKI, cardiac surgery associated acute kidney injury.

**eTable 3.** Discordance of hemodynamic variations in AKI cases based on different CSA-AKI definition criteria.

|  | **AKI defined by SCr only** | **AKI defined by UO only** | **AKI defined by SCr+UO** | ***P*-value** |
| --- | --- | --- | --- | --- |
| **Control** | 36 (52.9) | 90 (57.3) | 24 (61.5) | 0.675 |
| **CLCVP** | 32 (47.1) | 67 (42.7) | 15 (38.5) |  |
| Time-weighted average CVP, mmHg (median (IQR)) | 9.5 (6.6, 11.0) | 7.3 (5.1, 9.71) | 9.6 (6.9, 10.9) | 0.003 |
| CVP≥10 mmHg, n (%) | 49 (84.5) | 102 (70.8) | 29 (85.3) | 0.056 |
| Time-weighted average MAP, mmHg (median (IQR)) | 70.36 (65.2, 73.7) | 74.48 (69.2, 79.1) | 73.62 (67.8, 79.1) | 0.001 |

Abbreviations: CLCVP, controlled low central venous pressure; AKI, acute kidney injury; SCr, serum creatinine; UO: urine output; TWA, time weighted average; CVP, central venous pressure; IQR, interquartile range; MAP, mean arterial pressure.

**eFigure 1**. Incidence of acute kidney injury.

Abbreviations: AKI, acute kidney injury.

**eFigure 2.** Perioperative changes of serum creatinine relative to baseline values.

Abbreviations: SCr, serum creatinine.

**eFigure 3.** Postoperative changes of urine output among study subjects.
